# ZEAMAP, a Comprehensive Database Adapted to the Maize Multi-Omics Era

**DOI:** 10.1016/j.isci.2020.101241

**Published:** 2020-06-06

**Authors:** Songtao Gui, Linfeng Yang, Jianbo Li, Jingyun Luo, Xiaokai Xu, Jianyu Yuan, Lu Chen, Wenqiang Li, Xin Yang, Shenshen Wu, Shuyan Li, Yuebin Wang, Yabing Zhu, Qiang Gao, Ning Yang, Jianbing Yan

**Affiliations:** 1 National Key Laboratory of Crop Genetic Improvement, Huazhong Agricultural University, Wuhan 430070, China; 2 BGI Genomics, BGI-Shenzhen, Shenzhen 518083, China

**Keywords:** Plant Genetics, Bioinformatics, Plant Bioinformatics, Plant Biology

## Abstract

As one of the most extensively cultivated crops, maize (*Zea mays* L.) has been extensively studied by researchers and breeders for over a century. With advances in high-throughput detection of various omics data, a wealth of multi-dimensional and multi-omics information has been accumulated for maize and its wild relative, teosinte. Integration of this information has the potential to accelerate genetic research and generate improvements in maize agronomic traits. To this end, we constructed ZEAMAP, a comprehensive database incorporating multiple reference genomes, annotations, comparative genomics, transcriptomes, open chromatin regions, chromatin interactions, high-quality genetic variants, phenotypes, metabolomics, genetic maps, genetic mapping loci, population structures, and populational DNA methylation signals within maize inbred lines. ZEAMAP is user friendly, with the ability to interactively integrate, visualize, and cross-reference multiple different omics datasets.

## Highlights

• Functional annotations and comparative genomics of maize and teosinte genomes
• Multi-omics data generated from the same maize inbred lines panel
• Interactive tools to query, cross-refer, and visualize the omics data

## Graphical Abstract

**Figure:**
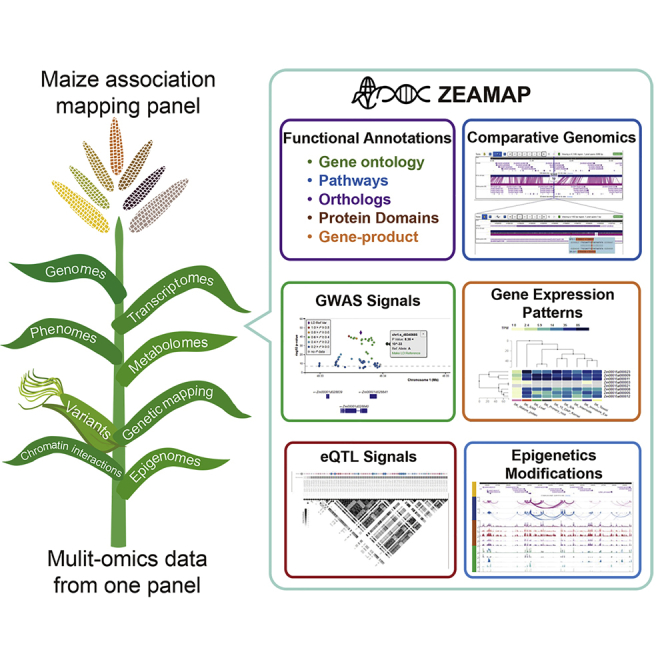

## Introduction

Maize (*Zea mays* L.) is one of the most important crops for food, feed, and fuel and is also a model species for genetic and genomic researches. As the cost of sequencing has been decreased and new omics technologies have arisen, there has been an explosive growth in the amount of biological information available for maize. The maize B73 reference genome has recently been updated (Jiao et al., 2017), and four high-quality maize genome assemblies have been released during the last 2 years (Li et al., 2019, Springer et al., 2018, Sun et al., 2018, Yang et al., 2019). The previous two-dimensional genome has recently been resolved in three dimensions with the mapping of open chromatin and the identification of chromatin interactions based on ChiA-PET and Hi-C technologies (Peng et al., 2019, Rodgers-Melnick et al., 2016). Omics data, including deep DNA resequencing, transcriptome, and metabolome, have been accumulated at the population scale (Hirsch et al., 2014, Hu et al., 2018, Li et al., 2013, Walley et al., 2016, Wang et al., 2018, Wen et al., 2014, Xu et al., 2019a, Zhou et al., 2019). There are many different applications for these new datasets, including gene cloning and the study of regulatory networks. These new and comprehensive datasets provide valuable resources for maize research and have the potential to completely revolutionize breeding (Wallace et al., 2018).

Comprehensive databases are needed to store, maintain, and analyze the multi-omics data that are now available for maize. Several maize genomics and functional genomics databases have been developed, including the Maize Genetics and Genomics Database (MaizeGDB) (https://www.maizegdb.org/), which collects maize reference sequences, stocks, and phenotypic and genotypic data and also provides useful tools for maize data mining (Lawrence et al., 2004, Portwood et al., 2018). Panzea (https://www.panzea.org/) collects genotypic and phenotypic information for several maize populations (Zhao et al., 2006), whereas MaizeNet (http://www.inetbio.org/maizenet/) provides a genome-scale co-functional network of maize genes (Lee et al., 2019). Other generic databases such as GenBank (https://www.ncbi.nlm.nih.gov/genbank/), Gramene (http://www.gramene.org/), and ePlant (http://bar.utoronto.ca/eplant_maize/) also collect maize omics data. Despite being very useful, these databases are designed either to collect general maize genomic and genetic information or to focus on one specific omics area. To make the best use of the multi-omics information for maize research and breeding, researchers currently need to either systematically integrate omics data generated from different sources (Rajasundaram and Selbig, 2016) or use multi-omics data that were all generated from the same panel.

MODEM (http://modem.hzau.edu.cn/) is the first attempt to integrate multi-omics datasets, including various types of genetic variants, expression data, and metabolomic data (Liu et al., 2016). Despite the importance of wild relatives in understanding the domestication of modern crops, no existing maize multi-omics databases incorporate teosinte (Tian et al., 2019). To fill this gap, we have developed ZEAMAP (http://www.zeamap.com/), a multi-omics database for maize research and breeding, which integrates omics data generated from 507 elite inbred lines (in an association mapping panel, AMP) (Yang et al., 2011) and 183 teosinte accessions (unpublished data). ZEAMAP includes genome assemblies and annotations of four inbred lines, B73 (Jiao et al., 2017), Mo17 (Sun et al., 2018), SK (Yang et al., 2019), and HuangZaoSi (HZS) (Li et al., 2019), and a teosinte accession (*Zea mays* ssp. *mexicana*) (Yang et al., 2017), expression patterns of tissues from different developmental stages of the same inbred line (Walley et al., 2016, Yang et al., 2019) and same tissue of different samples within the AMP (Li et al., 2013), three dimensional chromatin interactions and open chromatins of B73 (Peng et al., 2019), genetic variations including single-nucleotide polymorphisms (SNPs), small insertions and deletions (InDels) and large structure variations (SVs) generated from the deep sequencing of the AMP and the comparison among reference genome assemblies, the phenotypes and metabolome of the AMP and the related loci mapped by genome-wide association studies (GWASs), expression quantitative trait locus (eQTL) and linkage analysis, the population structure and pedigrees of each germplasm, and the populational DNA methylation signals within maize inbred lines. ZEAMAP generated comprehensive functional annotations for the annotated gene models in each assembly and provided useful tools for users to search, analyze, and visualize all these different omics data.

## Results

### Overview Structures of ZEAMAP Database

**Figure 1:**
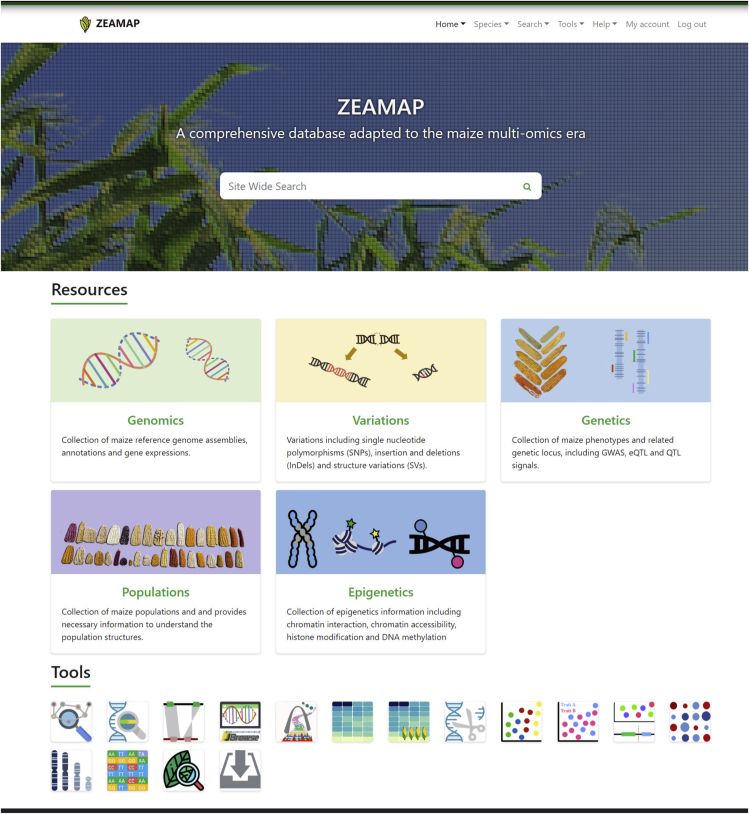
A Screenshot of the ZEAMAP Home Page The home page of ZEAMAP consists of a top menu bar, a site-wide search engine, access to the six biological modules, and miscellaneous tools.

ZEAMAP comprises a user account management system, a main database, a full-site search engine, and a set of analysis and visualization tools (Figure S1). The multi-omics data in ZEAMAP are categorized into five main content modules involving genomic, genetic, variation, population, and epigenetic information (Figures 1 and S1). ZEAMAP construction utilized the biological community database construction toolkit Tripal (Ficklin et al., 2011), which combines the content management system Drupal (https://www.drupal.org) with the standard biological relational database storage backend, Chado (Jung et al., 2016). Each feature in ZEAMAP has its own page, and features are linked to each other by sequence ontology relationships.

### The Genomics Module

The Genomics module collects reference genome assemblies, gene expression profiles, and comparative genomics information related to the available genomes and populations in ZEAMAP. Currently, ZEAMAP contains reference genome assemblies of four maize inbred lines (B73, SK, Mo17, and HZS) and one teosinte species (*Zea mays* ssp. *mexicana*). Each genome assembly has its own page, which contains general information for each genome assembly and sub-menus with links to access various related information and bioinformatics analysis tools (Figure S2). The mRNA and predicted protein for gene models in each genome assembly were assigned functional annotations including gene ontologies (GO), Kyoto Encyclopedia of Genes and Genomes pathways (KEGG), clusters of orthologous groups (COG), orthology relationships, Single Copy Orthologs (SCO), known gene-product annotations, proteolytic enzymes, and related items in InterPro, PFAM, and NCBI nr databases (Figure S3). The genome features for each assembly, including genes, mRNAs, proteins, and transposable elements, can be searched by their IDs or locations through Chado feature search (Figure S4A). Genes (as well as mRNAs and proteins) can also be searched by their functional annotation descriptions (Figure S4B). Each annotated genome feature has its own page with multiple sub-menus displaying summary information (resource type, accession, organism, name, identifier, and others), sequences, annotations, cross-references linked to the same feature in MaizeGDB or NCBI, as well as related parent and child features, orthologs, and synteny blocks.

**Figure 2:**
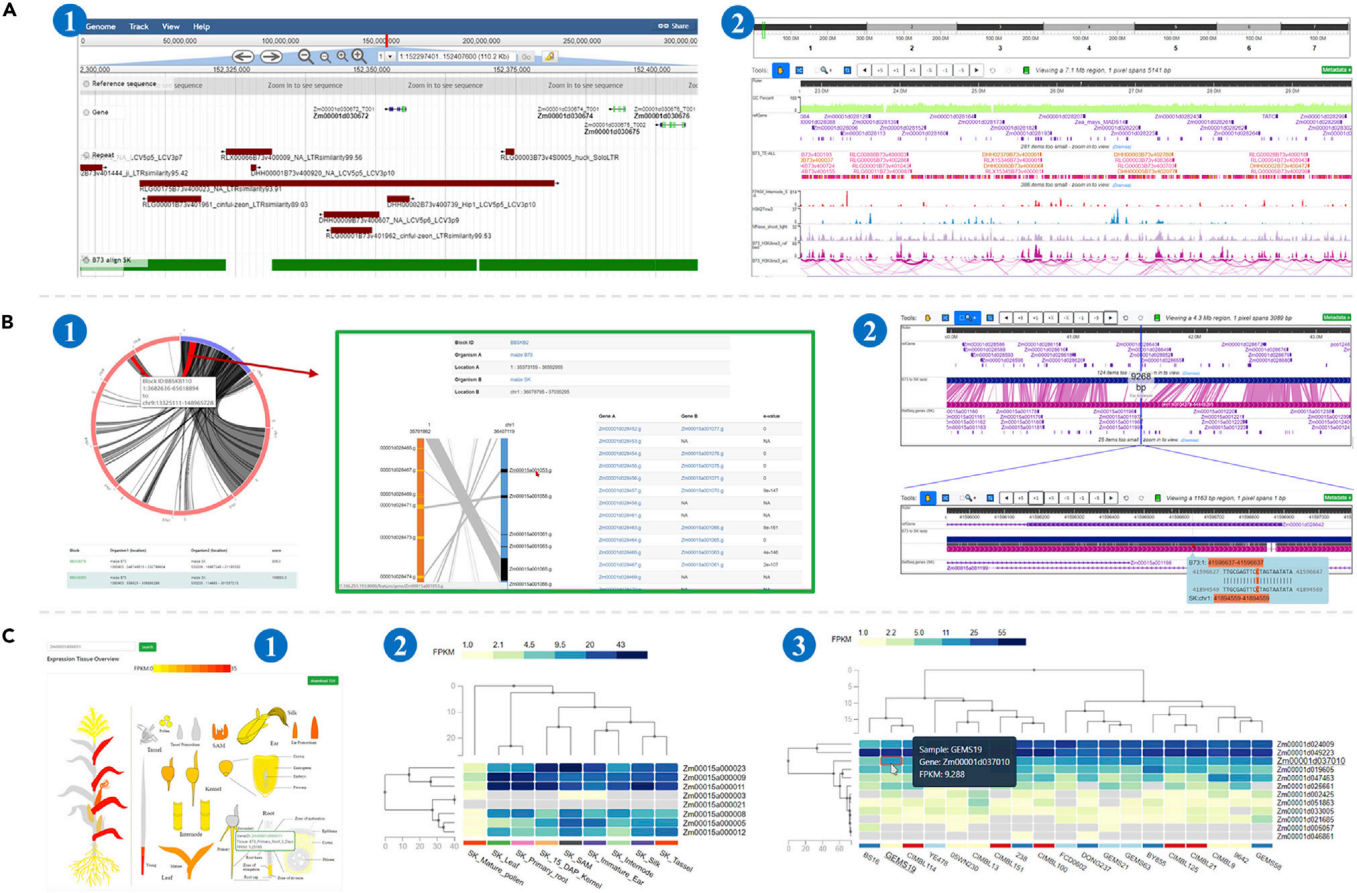
Features of the ZEAMAP Genomics Module (A) Schematic diagram of the two genome browsers embedded in ZEAMAP, Jbrowse(1) and the WashU Epigenome Browser(2). (B) Comparative genomic information in ZEAMAP, including Gene synteny blocks displayed by the synteny viewer with interactive circos plots and links to the detailed collinearity of the included genes (1). Whole-genome sequence alignments between two genomes are also accessible through the WashU Epigenome Browser, with Zoom-In and -Out functions and mouse-over display of the detailed alignments (2). (C) Gene expression functions in ZEAMAP. The Tissue Overview function shows the expression of a gene in different tissues, with more detailed information available upon click (1). ZEAMAP also has functions to cluster and display the expression patterns of several genes by tissue type (2) or sample (3), with the gene IDs linked to pages with more detailed information.

Two genome browsers, JBrowse (Buels et al., 2016) and WashU Epigenome Browser (Zhou et al., 2013), were embedded to display the genome sequences, annotated genomic features, and other genomic information for all the available reference genome assemblies in ZEAMAP (Figure 2A). Both genome browsers are designed to easily add tracks, search for certain information in specific regions, and export data as well as figures. These two genome browsers share some common features, including genome sequences and genomic annotations. However, each one has unique information tracks (see sections below) because JBrowse performs better when dealing with large piecemeal features such as variations and the WashU Epigenome Browser is specially designed to display epigenomic tracks.

ZEAMAP provides comparative genomic information for each pair of the available reference genome assemblies, including both synteny blocks identified from gene collinearities and whole-genome alignment details. The synteny blocks are managed and displayed through the Tripal synteny viewer module (https://github.com/tripal/tripal_synview). Each synteny block has its own unique block ID and can be searched by it in both the Synteny block browser page and in the full-site search engine (Figure 2B). The detailed whole genome sequence alignments can also be accessed through the genome browser (Figure 2B).

We have collected gene expression patterns in different tissues for each maize genome assemblies, as well as expression profiles of kernels for 368 inbred lines of the AMP (Li et al., 2013) based on B73 reference annotations. Expression patterns in different tissues for each gene can be visually displayed through heatmaps after being queried in the “Tissue Overview” page (Figure 2C). ZEAMAP also enables users to browse the expression patterns of several genes among different tissues or samples and cluster the genes and tissues/samples based on the gene expression patterns (Figure 2C). Both functions provide download links to a raw expression matrix of the queries.

### The Variations Module

The Variations module collects the genotypes and annotations of polymorphic variations including SNPs, InDels, and SVs among the AMP in reference to the B73 reference genome, as well as a haplotype map generated from the SNP genotype matrix (see Transparent Methods for the source and the analysis used to generate the related data). The general variation information of a gene, including variation positions, allele types, and annotations can be queried by their IDs or locations and displayed through tabular view (Figure S5A). The variations can also be browsed through JBrowse. Upon clicking each variant block in JBrowse tracks, the detailed information about that variant, including the annotations and the genotype of each germplasm, will be shown. There is also a genotype overview for the variations in the current JBrowse display panel when the related “variant matrix” track is selected (Figure S5B). ZEAMAP also provides a function to query for the detailed genotype matrix for specified germplasms within certain regions (Figure S5C).

### The Genetics Module

**Figure 3:**
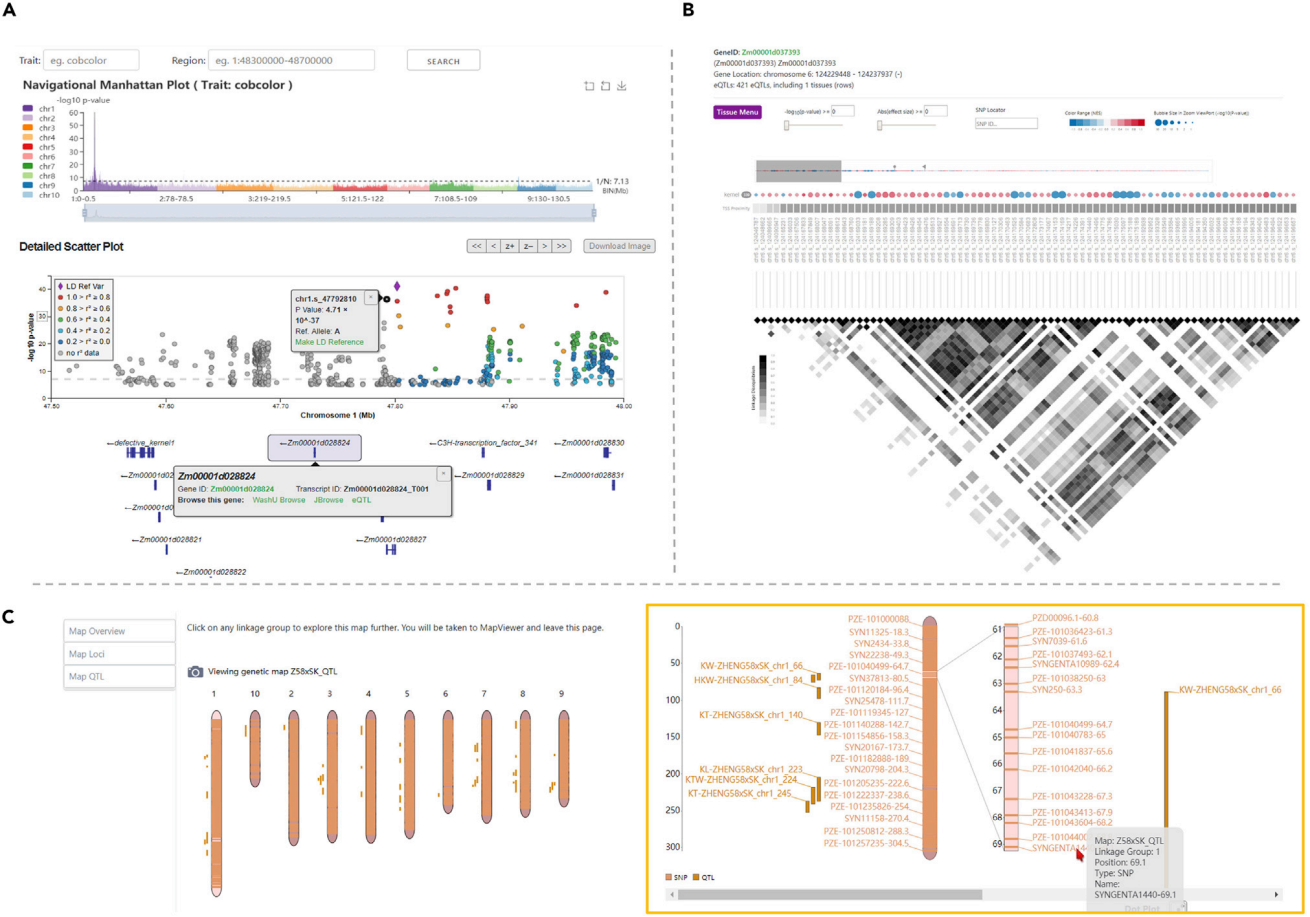
Features of the ZEAMAP Genetics Module (A) Schematic diagram of the “GWAS-Single-Trait” tool. The trait and region of interest can be queried through the top input boxes. Regions can be easily browsed by clicking on the histogram of the interactive “Navigational Manhattan Plot” track. The “Detailed ScatterPlot” track plots the variants according to their chromosome locations and by the significance of their *p* values. The colors of each dot indicate the LD r^2^ values between that variant and the reference variant (the purple diamond dot, can be reset by selecting the “Make LD reference” link on the popup page for each variant). The bottom track shows the gene annotations in the selected region, with a popup for each gene element that links to a detailed information page, genome browsers, and the eQTL visualizer for that gene. (B) Schematic diagram of the eQTL visualization tool. The significant *cis*-eQTL site for each gene is sized by the significance of its *p* value and colored by the effect size (beta value). The heatmap indicates pairwise LD r^2^ values of the variants. (C) Schematic diagram of the TripalMap tool in ZEAMAP. This tool displays the detailed genetic markers and mapped QTLs for each linkage group. Both the markers and the QTLs link to their own detailed information page.

ZEAMAP has collected phenotypic data from the AMP, including 21 agronomic traits, 31 kernel lipid content-related traits, 19 kernel amino acid content-related traits, and 184 known metabolites of maize kernels. All these phenotypes can be searched and filtered by their threshold values using the “Search Trait Evaluation” tool (Figure S6). We have identified loci significantly associated with these phenotypes using GWAS and provided a tabular data search function to find specific loci by trait names, variant IDs, chromosome regions, and significant *p* values (Figure S7). Three GWAS visualization tools (“GWAS-Single-Trait,” “GWAS-Multi-Trait,” and “GWAS-Locus”) were developed to better browse the GWAS results and compare the significant signals among different traits. Querying a trait and navigating to specific regions can be easily accomplished by inputting boxes or through the interactive navigational Manhattan plot (in GWAS-Single-Trait and GWAS-Multi-Trait tools). The GWAS-Single-Trait tool displays all signals associated with the selected trait as a scatterplot, with colors indicating the linkage disequilibrium (LD) r^2^ values between the user-selected reference variant and all the other variants (Figure 3A). The GWAS-Multi-Trait tool was designed to compare GWAS signals among two or more traits, with the colors indicating different traits (Figure S8), whereas the GWAS-Locus tool displays GWAS signals of all traits that show significant association with the query variant (Figure S9). These three tools are provided with a lightweight genome browser that indicates the gene models within the current region. Each element in the plot is also interactive and links to other related information.

Genetic variations can impact gene expression through many factors, including alterations in splicing, noncoding RNA expression, and RNA stability (Gilad et al., 2008). Expression quantitative trait locus (eQTL) mapping is a powerful approach to detect the possible variants that alter gene expression. In ZEAMAP, we have collected *cis*-eQTL signals with gene expression patterns in maize kernels based on B73 annotation and provided a tabular tool to search and filter eQTL signals by gene IDs, gene locations, distances from transcription start site (TSS), effect sizes, and significance values (Figure S10). A visualization tool was also developed to browse all *cis*-eQTLs affecting the selected gene, with significance values, effect size, and pairwise LD information displayed interactively (Figure 3B).

ZEAMAP has currently collected 12 published genetic maps constructed from different artificial maize segregating populations using genotypes generated from the Illumina MaizeSNP50 BeadChip (Illumina, San Diego, CA, USA), as well as 813 quantitative trait loci (QTLs) identified from 15 plant architecture-related traits (Pan et al., 2016). The genetic markers can be searched and filtered by their IDs, genomic locations, and genetic linkage group (Figure S11). QTLs can be searched by traits and QTL labels, resulting in detailed records of the genetic markers located in or adjacent to that QTL. By employing the TripalMap extension module (https://github.com/ksbuble/TripalMap), the linkage maps, including all related markers and QTLs, can be visualized and compared with another map interactively (Figure 3C).

### The Populations Module

**Figure 4:**
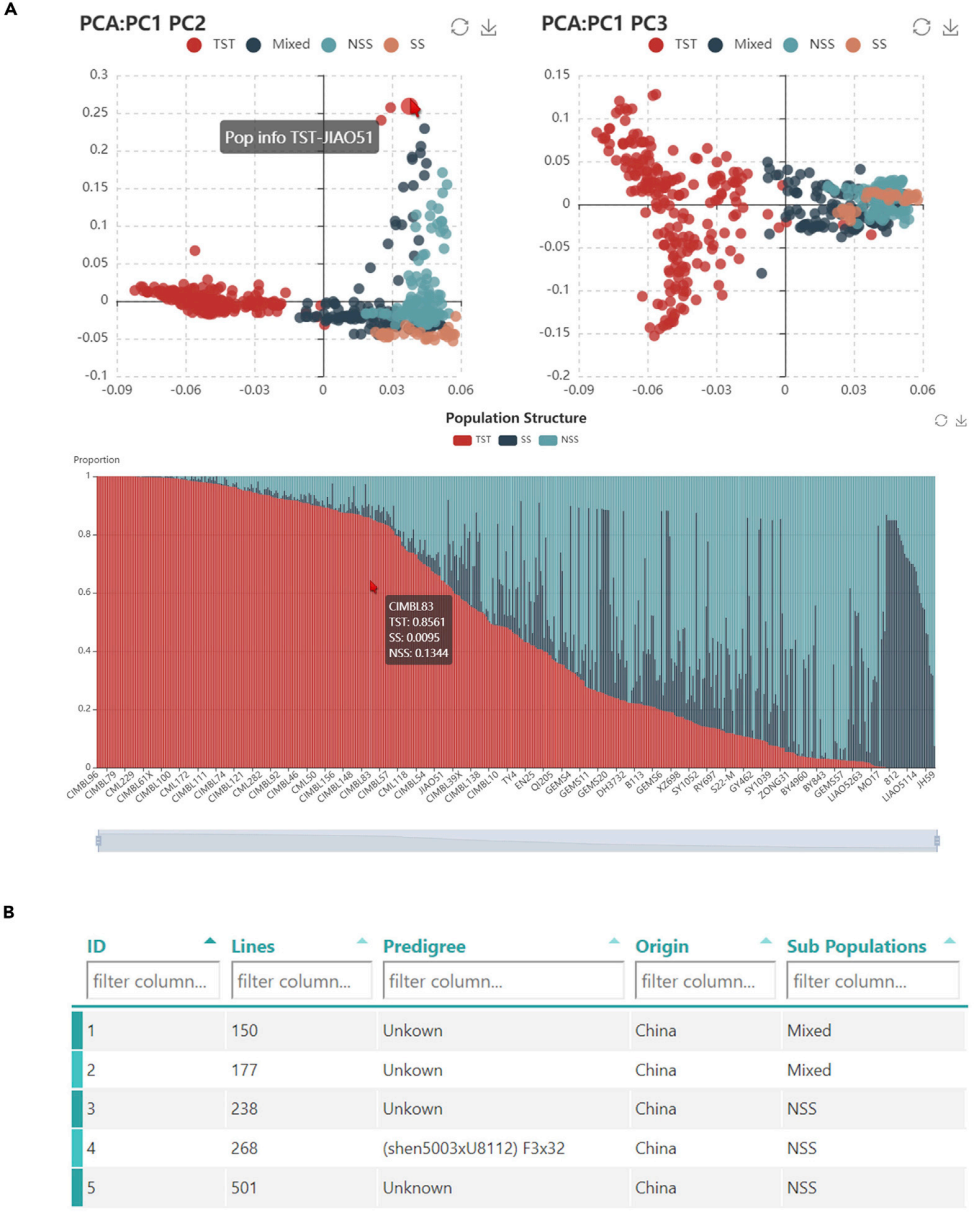
Features of the ZEAMAP Populations Module (A) Interactive PCA diagram (top two dot plots) and structure diagram (stacked bar plot). Each diagram is zoomable and shows detailed information, including germplasm names and PCA/structure values when an element is moused over. (B) A table browser is provided to search for germplasm by pedigree, origin, and subpopulation information.

It is often useful to dissect the genetic diversity, population structure, and pedigrees of maize lines for both evolutionary studies and molecular breeding. ZEAMAP provides interactive information about the population structures assessed by principal component analysis (PCA) and ancestries inferred from an unsupervised clustering analysis for the whole *Zea* population and each sub-population in the database (Figure 4A). We have also added a table that lists the origins or pedigree information for each inbred line of maize AMP (Figure 4B).

### The Epigenetics Module

**Figure 5:**
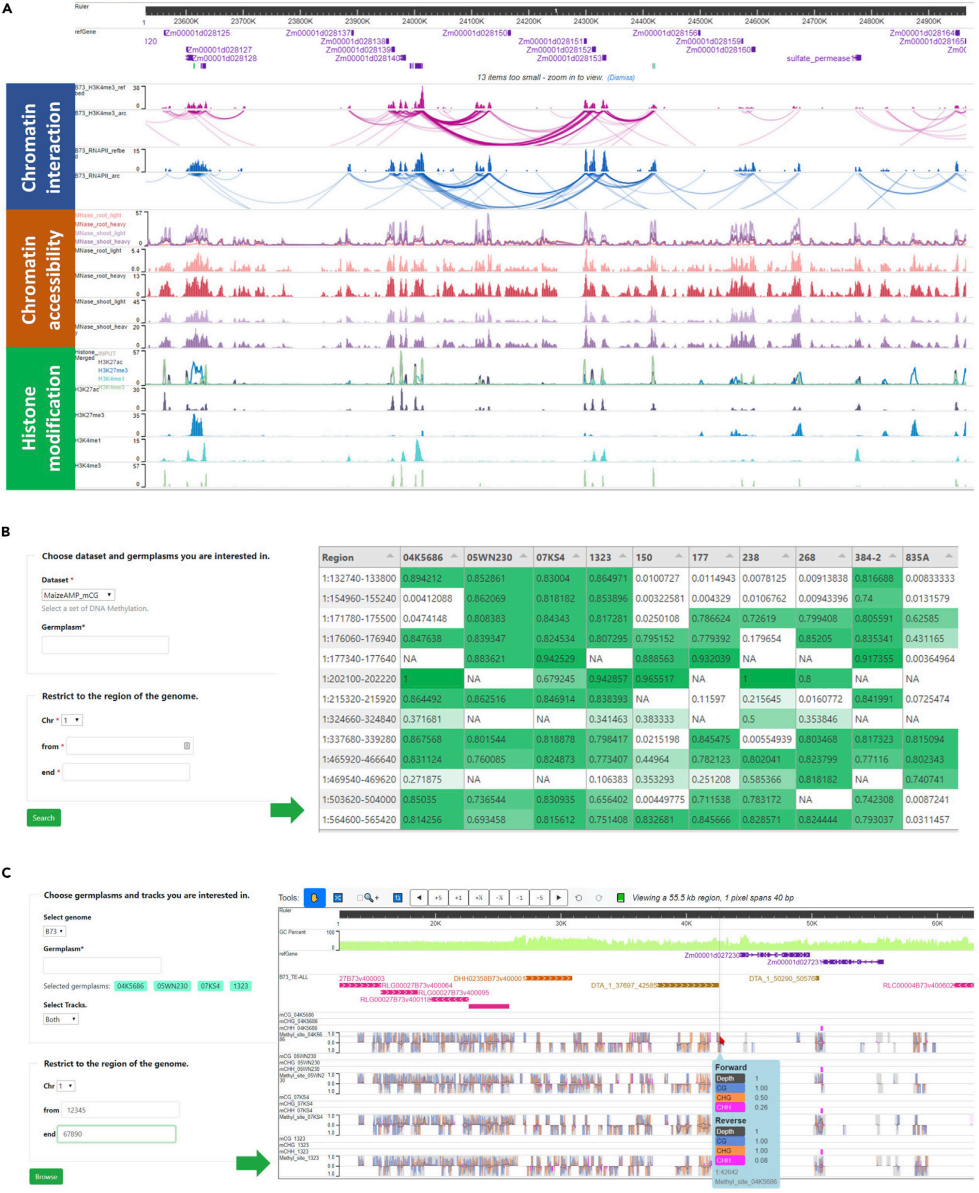
Features of the ZEAMAP Epigenetics Module (A) Schematic diagram of chromatin interaction, chromatin accessibility, and histone modification tracks displayed in the WashU Epigenome Browser. (B) Populational DNA methylation table browser. This tool filters population DNA methylation information by the DNA methylation type, germplasm, and genomic region of interest, with the resulting matrix displaying DMRs for each selected germplasm within the query region. (C) Interface of the population DNA methylation genome browser. This interface provides options to display DNA methylation information by DMRs or DNA methylation sites of the selected germplasms within specified regions.

Eukaryotic gene expression has been shown to be altered by three-dimensional DNA interactions, which are affected by chromatin accessibility. Additionally, the modifications of epigenetic states on histones and nucleotides add another layer of control to gene expression regulation (Dekker, 2008). These regulatory factors are crucial for the ability of sessile plants to respond to diverse environmental challenges (He and Li, 2018). In ZEAMAP, we have collected the chromatin interaction maps associated with RNA polymerase II occupancy and the histone mark H3K4me3 according to the B73 reference genome (Peng et al., 2019). Open chromatin regions are based on micrococcal nuclease (MNase) digestion (Rodgers-Melnick et al., 2016), histone acetylation and methylation regions, and populational DNA methylation information generated from the third leaves at V3 of the 263 AMP inbred lines (Xu et al., 2019b). This information can be accessed through a tabular data browser or visualized through the WashU Epigenome Browser (Figure 5A). For DNA methylation information from the AMP, customized interfaces were developed to easily select multiple samples with differentially methylated regions (DMRs) in the table browser (Figure 5B) and visualize both DMR and DNA methylation sites in the WashU Epigenome Browser (Figure 5C).

### Additional Tools

In addition to the aforementioned major biological modules, ZEAMAP also offers several additional tools. The currently available additional tools include a site-wide search engine, BLAST server, a CRISPR browser and an FTP data downloader.

**Figure 6:**
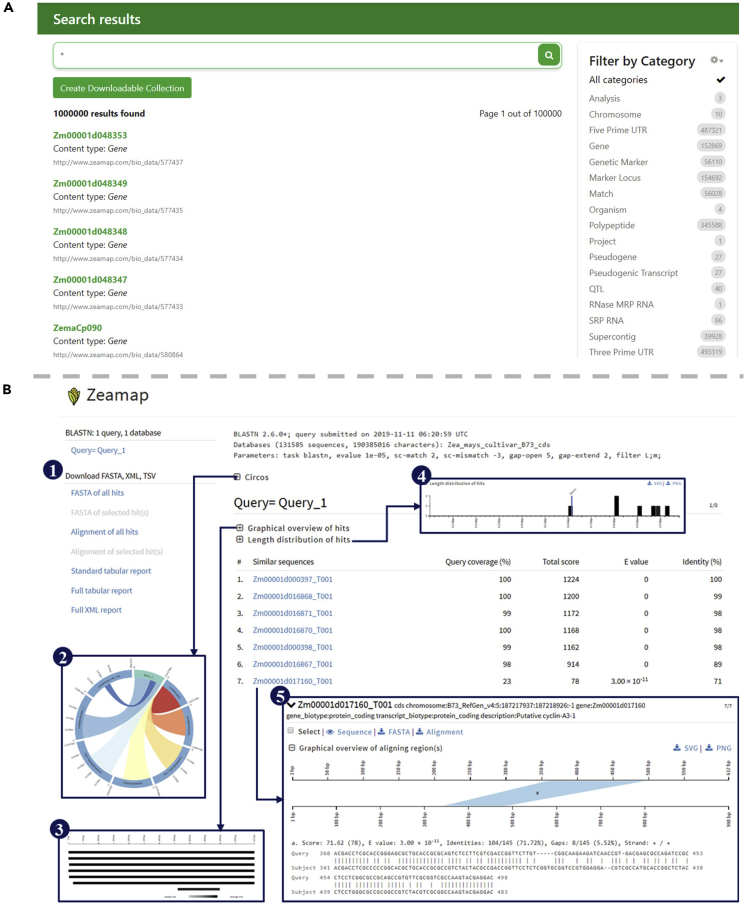
Features of the Additional Functional Tools in ZEAMAP (A) An example search result by the site-wide search engine queried with an asterisk wildcard. The resulting items are categorized by their feature types and have links to their detailed information pages. (B) An example result of the BLAST server in ZEAMAP. The result page provides download links of sequences (1) with reports available in different formats. Also included are interactive plot views of each alignment including Circos plots (2), NCBI BLAST-like alignment hits visualization (3), and length distribution of hits (4). Each alignment hit has detailed alignment information, including a graphic view of the aligned regions and detailed alignments (5).

Although there are already independent search tools for several of these analyses, a site-wide search engine powered by Chado is still useful since it enables users to quickly search for all items related to their queries. The ZEAMAP site-wide search engine was built using the Tripal Elasticsearch module (https://github.com/tripal/tripal_elasticsearch), with a search box that is accessible in both the home page and the status bar of each page. The search engine supports advanced search behaviors including wildcards, fuzzy searches, regular expressions, and Boolean operators. The search results are also categorized by their entity type in the database (Figure 6A).

We have also implemented an instance of NCBI's BLAST tool in ZEAMAP using SequenceServer (Priyam et al., 2019), which provides a user-friendly interface with text-based and interactive visual outputs (Figure 6B). ZEAMAP currently has BLAST databases for whole-genome sequences, mRNAs, CDSs, and predicted proteins for each reference genome assembly.

ZEAMAP also includes an imbedded tool to search for reliable single-guide RNAs (sgRNAs) targeting the genes in each maize genome assembly in the database in order to support genome editing experiments using the CRISPR-Cas9 system. The sgRNA information can be browsed tabularly when querying a gene ID or a genomic region and graphically through JBrowse. Both the tabular and the graphical results provide information about the editing positions and possible off-target genes (Figure S12).

Additionally, we have provided an FTP server to store a backup of all the publicly released datasets used in ZEAMAP through h5ai (https://larsjung.de/h5ai/), an open source file indexer with an enhanced user interface, text preview, and directory download.

## Discussion

We have created ZEAMAP, a database for maize research and breeding that collects multi-dimensional omics information, including genome assemblies, comparative genomics, transcriptomes, open chromatin, chromatin interactions, genetic variants, phenotypes, metabolomics, genetic maps, genetic mapping loci, population structures and pedigrees, and populational DNA methylation signals within maize inbred lines. Most of the datasets were generated from the same maize population, which makes it possible to cross-reference these multi-omics data to support maize research in a more uniform and comprehensive manner. To make the acquisition and analysis of information more effective and flexible, ZEAMAP provides several convenient modules, including a site-wide search function, dataset-specific search tools, a BLAST server, a gene expression pattern analyzer, tabular browsers, genome browsers, and specialized visualizers for different datasets. ZEAMAP will be carefully maintained and continuously updated with the latest genomic and genetic advances. More online analysis tools (software for LD and PCA analyses, for example) will be embedded in ZEAMAP in the near future. Ultimately, we plan to systematically integrate all available omics data and make ZEAMAP a platform to analyze relationships between genotypes and phenotypes in order to predict complex traits for maize researchers and breeders.

### Limitations of the Study

Currently, ZEAMAP has mainly focused on collection, query, and visualization of pre-analyzed datasets, with only several lightweight online analysis tools embedded. The lack of comprehensive online analysis tools that make the best of the multi-omics data in ZEAMAP to help users better understand their custom data is the main limitation for the current version of ZEAMAP database.

### Resource Availability

#### Lead Contact

Further information and requests for resources and reagents should be directed to and will be fulfilled by the Lead Contact, Ning Yang (yangningyingji@126.com).

#### Materials Availability

This study did not generate new unique reagents.

#### Data and Code Availability

All the strategies and data included in this paper are available from ZEAMAP (http://www.zeamap.com). The related source codes are available at https://github.com/ZEAMAP.

## Methods

All methods can be found in the accompanying Transparent Methods supplemental file.

## Supplemental Information

Document S1. Transaparent Methods and Figures S1–S12
