# Supplementary material for: ZEAMAP, a Comprehensive Database Adapted to the Maize Multi-Omics Era
Source: iScience. 2020 Jun 6;23(6):101241. doi: 10.1016/j.isci.2020.101241 (PMC7306594; doi:10.1016/j.isci.2020.101241)
Supplement: Document S1. Transaparent Methods and Figures S1–S12 [file mmc1.pdf]

iScience, Volume *23*

Supplemental Information

ZEAMAP, a Comprehensive Database Adapted to the Maize Multi-Omics Era

Songtao Gui, Linfeng Yang, Jianbo Li, Jingyun Luo, Xiaokai Xu, Jianyu Yuan, Lu Chen, Wenqiang Li, Xin Yang, Shenshen Wu, Shuyan Li, Yuebin Wang, Yabing Zhu, Qiang Gao, Ning Yang, and Jianbing Yan

Supplemental Files

Supplemental Figures

Figure S1. Sketch of ZEAMAP database structure.

Figure S2. Species detailed page of *Zea mays* cultivar B73.

Figure S3. Functional annotation information in each gene's detailed page.

Figure S4. Feature search and gene search functions in ZEAMAP.

Figure S5. Features of ZEAMAP variations module.

Figure S6. Search trait function in ZEAMAP.

Figure S7. GWAS table browser in ZEAMAP.

Figure S8. GWAS-Multi-Trait visualization tool in ZEAMAP.

Figure S9. GWAS-Locus visualization tool in ZEAMAP.

Figure S10. eQTL table browser in ZEAMAP.

Figure S11. Genetic marker search functions in ZEAMAP.

Figure S12. Crispr sgRNA function in ZEAMAP.

Transparent Methods

Supplementary References

Supplemental Figures

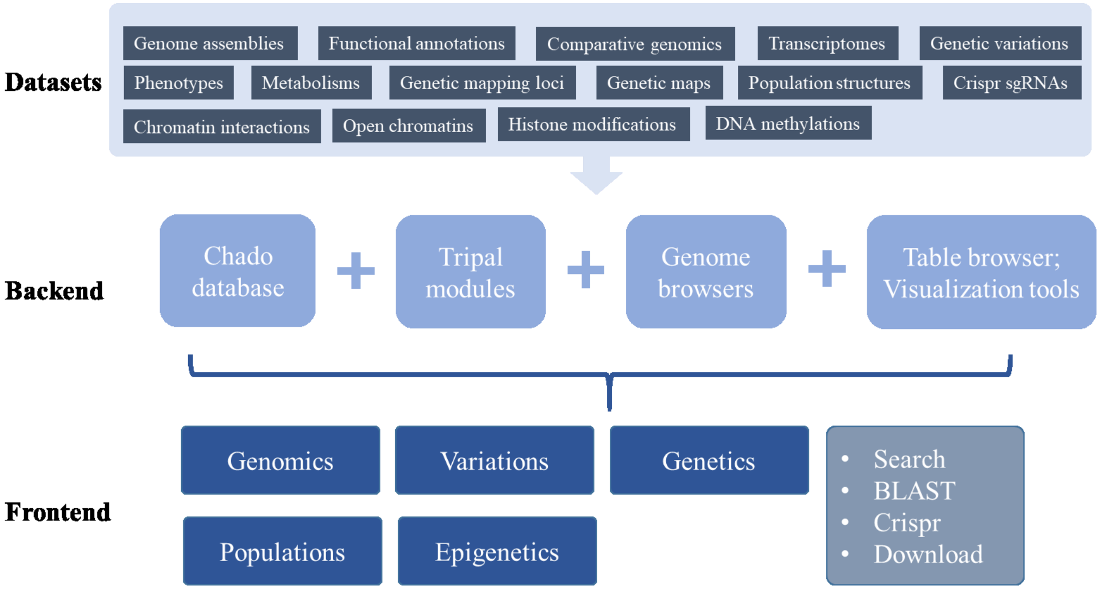

Figure S1. Sketch of ZEAMAP database structure, Related to Figure 1. The multi-omics data were stored into database and managed by chado sequence ontology and tripal modules. Two genome browsers, custom table browsers and visualization tools were developed for data querying and visualization. The frontend of ZEAMAP were divided into five main biological sections (Genomics, Variations, Genetics Populations and Epigenetics), and miscellaneous tools including site-wide search engine, BLAST server, Crispr sgRNA searcher and FTP Download server.

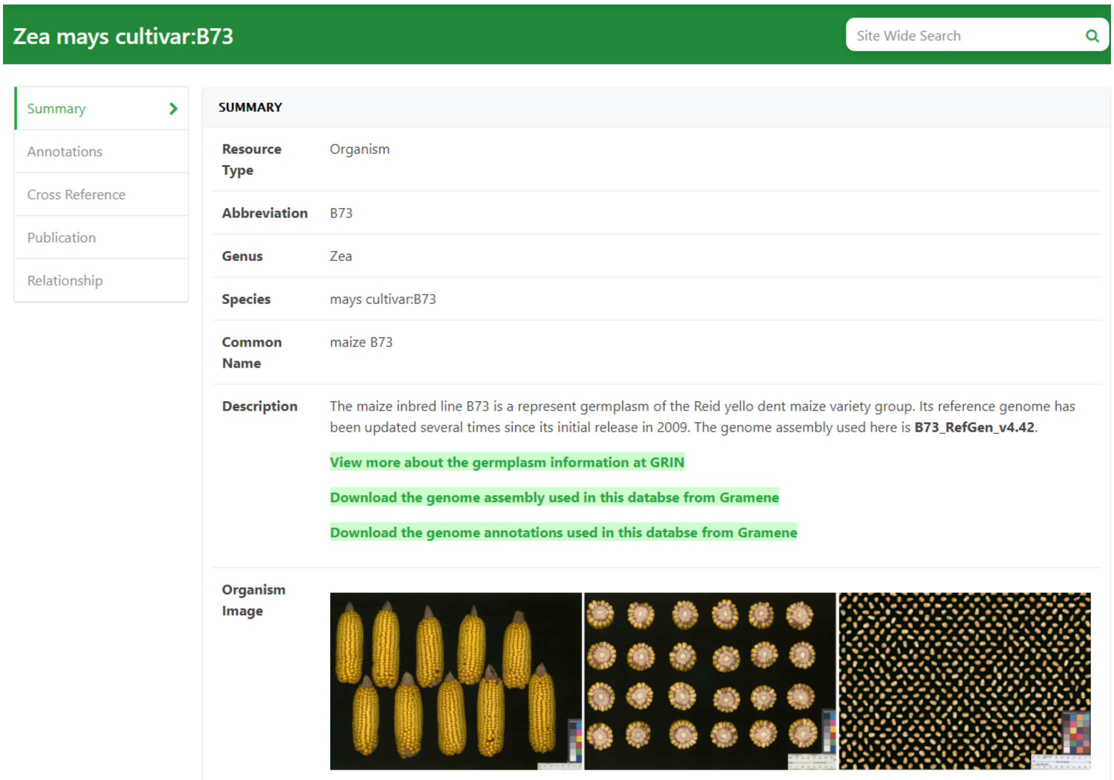

Figure S2. Species detailed page of *Zea mays* cultivar B73, Related to Figure 2. The species page shows the general information about the current species/germplasm, including brief introductions, external links to the germplasm information and the accessions of related omics data.

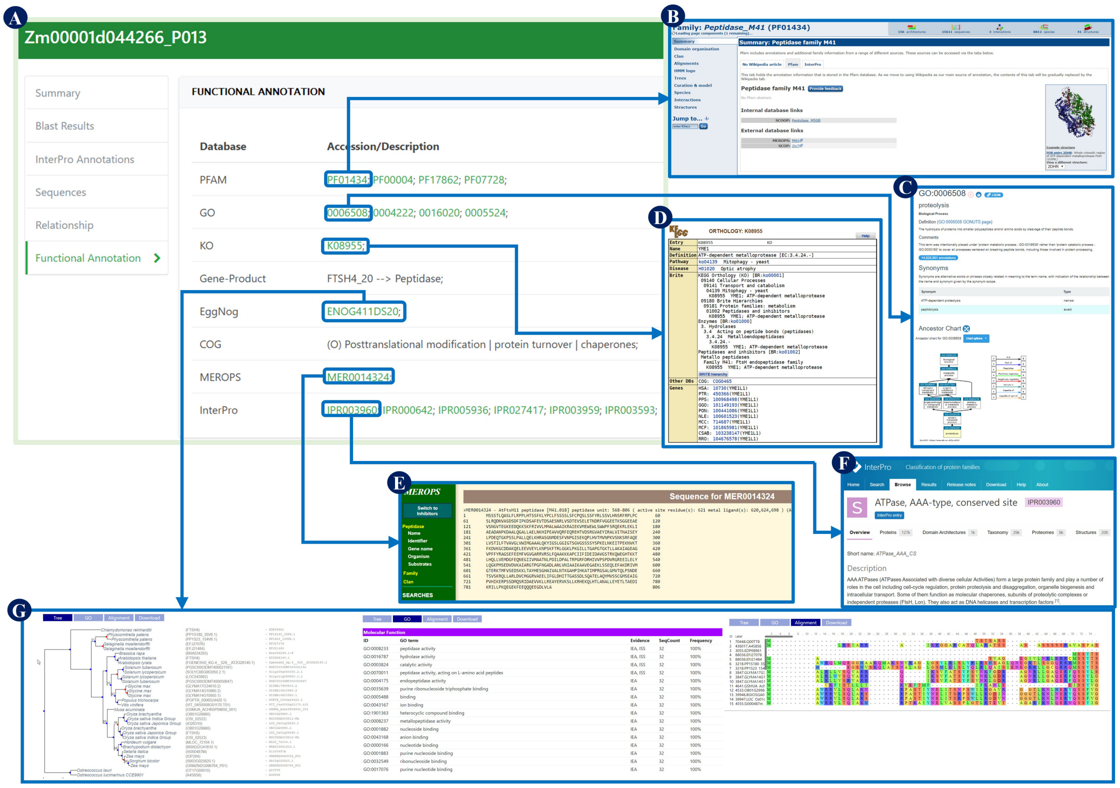

Figure S3. Functional annotation information in each gene's detailed page, Related to Figure 2. (A) The "Functional annotation" tag in the detailed page of each gene/mRNA/ protein (taking the detailed page of protein Zm00001d044266_P013 for example here), the annotated items within each database were linked to their detailed information page (B-F). A specific page was developed to get the detailed information of the related ortholog group including the phylogenetic relationships, the gene ontology items, and the sequence alignments within the ortholog group from EggNOG database (G).

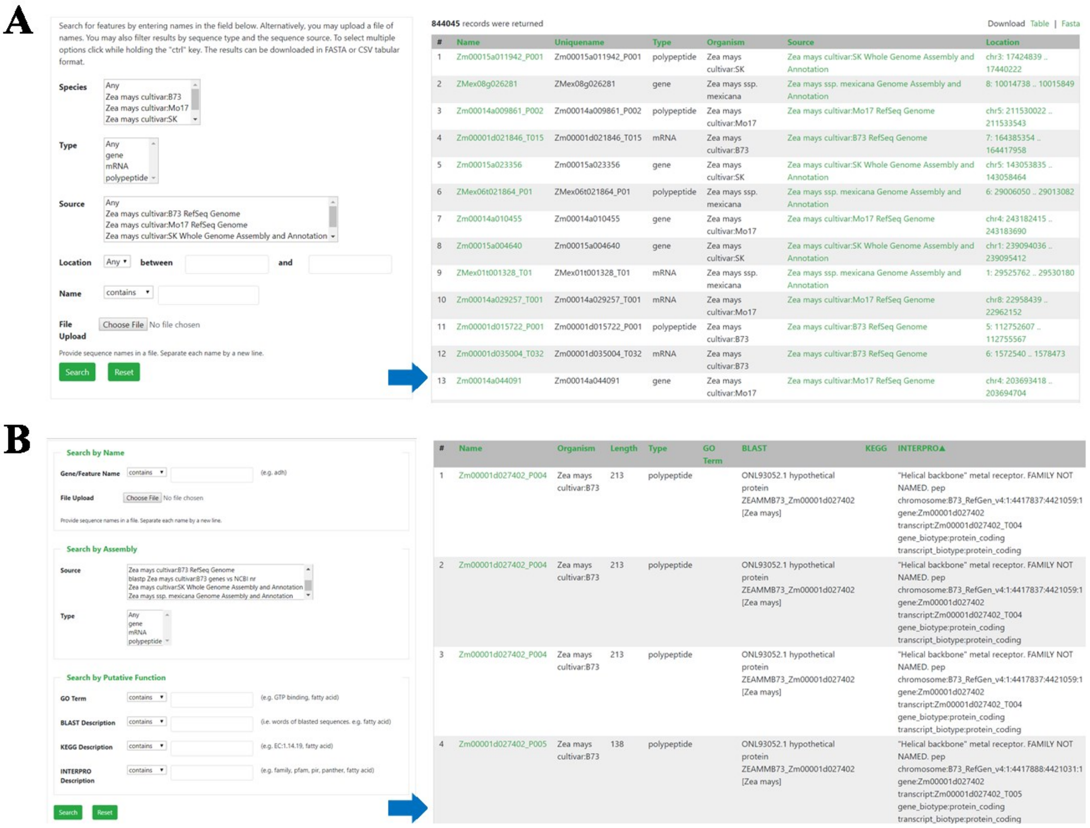

Figure S4. Feature search and gene search functions in ZEAMAP, Related to Figure 2. The feature search function (A) in ZEAMAP enables searching for all annotated features on certain genome assemblies by their locations and/or names. The locations of the search results were displayed and linked to genome browser for visualization. The gene search function (B) is dedicated to search for annotated genes, mRNAs and polypeptides by their names and/or their functional annotations, with the detailed functional annotations displayed in the search results for each record.

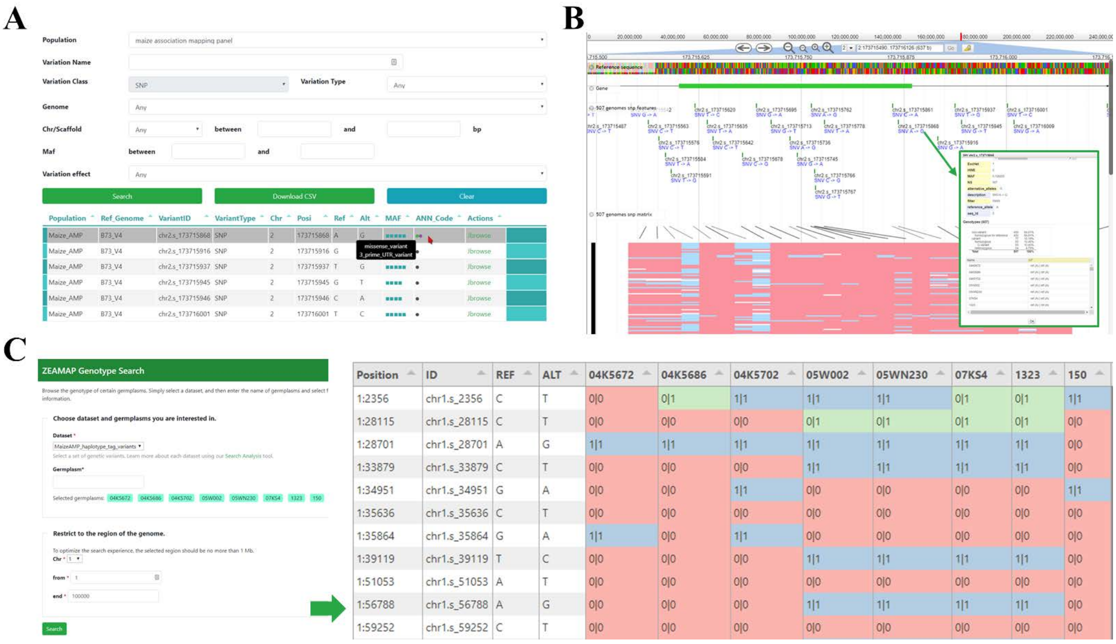

Figure S5. Features of ZEAMAP variations module, Related to Figure 1. In ZEAMAP, variants could be displayed tabularly and filtered by IDs, locations and annotated effects (A), with each resulted record has links to its location on Jbrowse (B). The popup for each variant in Jbrowse shows the detailed information including annotations and genotype in each sample (inset in B). Jbrowse has also provided an additional track shows an overview of genotype matrix (red: reference genotype; light blue: homozygous genotype; light pink: heterozygous; grey: no call). (C) ZEAMAP has also provided a “Genotype Search” function to get the genotype information by genomics regions and germplasms of interest, with a resulted matrix shows the variant positions, their IDs, reference and alternative alleles and genotypes in each samples (“0” for reference allele and “1” for alternative allele; red: homozygous reference genotype; blue: homozygous alternative genotype; green: heterozygous genotype).

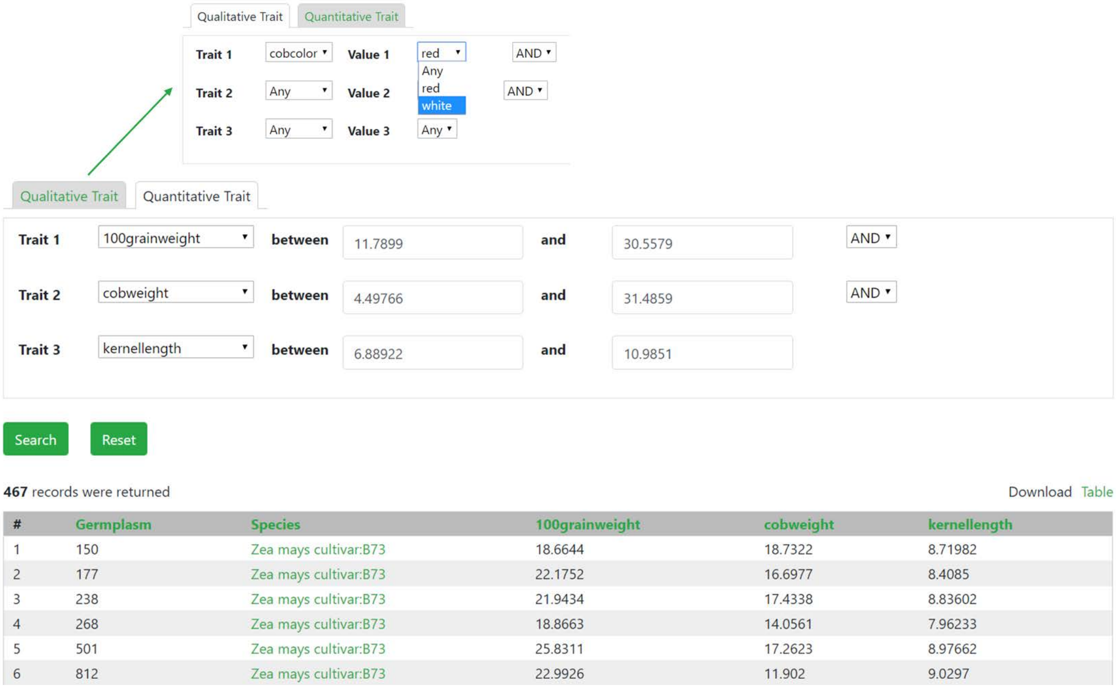

Figure S6. Search trait function in ZEAMAP, Related to Figure 3. Both qualitative and quantitative trait could be searched by their trait values with multiple filter conditions supported. The search result shows all the germplasms that passed the filter conditions and their triat values.

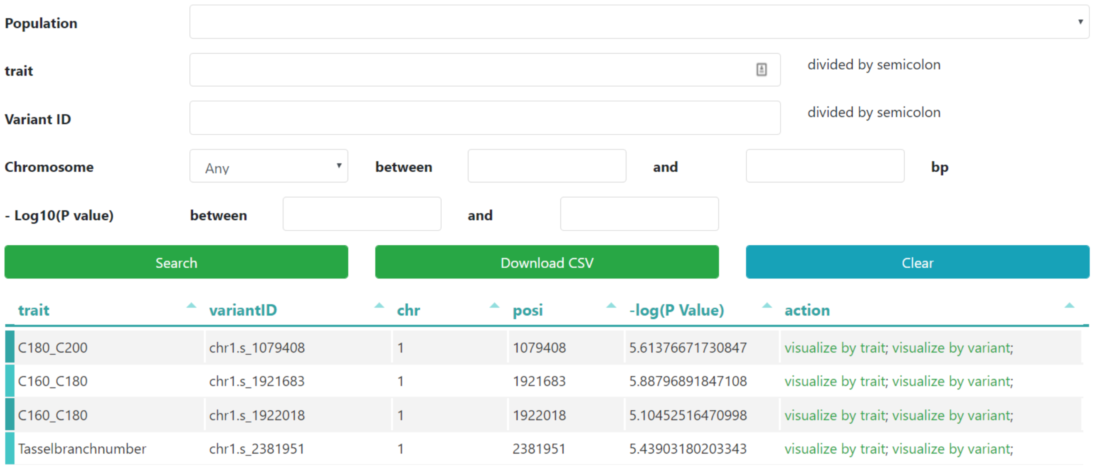

Figure S7. GWAS table browser in ZEAMAP, Related to Figure 3. The GWAS signals could be searched by traits, variant IDs and variant locations, and filtered by significant P values. Each record in the search result has links to the GWAS visualization tools.

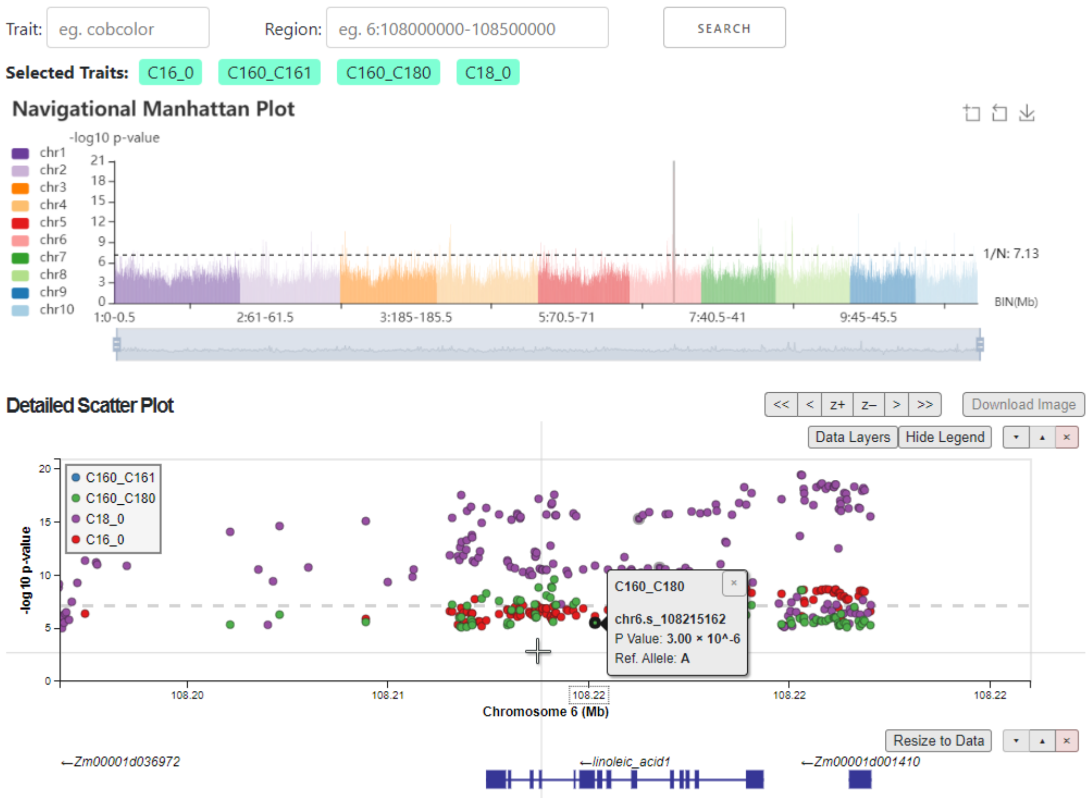

Figure S8. GWAS-Multi-Trait visualization tool in ZEAMAP, Related to Figure 3. This tools displays GWAS signals of multiple traits, with logic similar to GWAS-Single-Trait tool (as indicated in Figure 2A). The only differences are that the traits here are multi-selectable, and the colors in the detailed scatter plot indicate variants for different traits rather than LDs. A “data layers” button has been added in the control panel of the detailed scatter plot to fade, hide, order or remove certain trait layers.

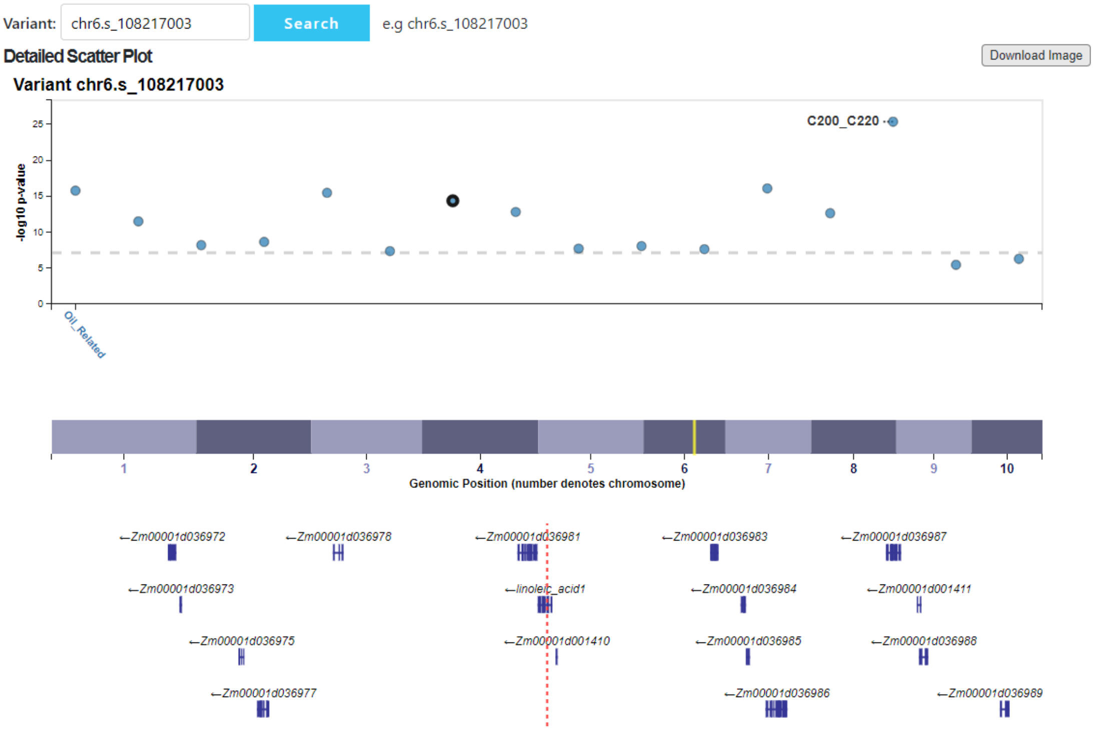

Figure S9. GWAS-Locus visualization tool in ZEAMAP, Related to Figure 3. This tool displays all significantly associated signals between the query variant and all available traits, with a yellow highlight line and a red dashed line respectively indicated the general and detailed position of the query variant.

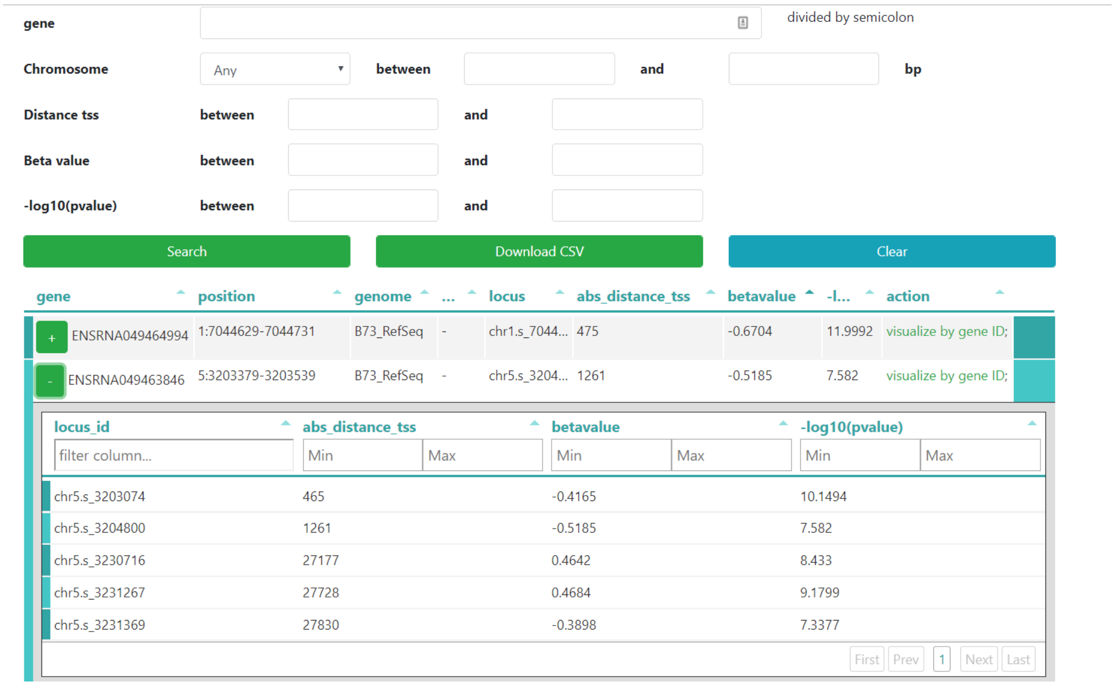

Figure S10. eQTL table browser in ZEAMAP, Related to Figure 3. Using this tool, eQTL signals could be filtered by gene IDs and locations, as well as the distance from transcription start site, the effect size (beta value) and the significance (p value) of the most significant variant within each gene. The search result shows one gene per record, with links to the visualization of each gene. Each record has a sub-table which lists all the *cis*-eQTL signals significantly associated with this gene.

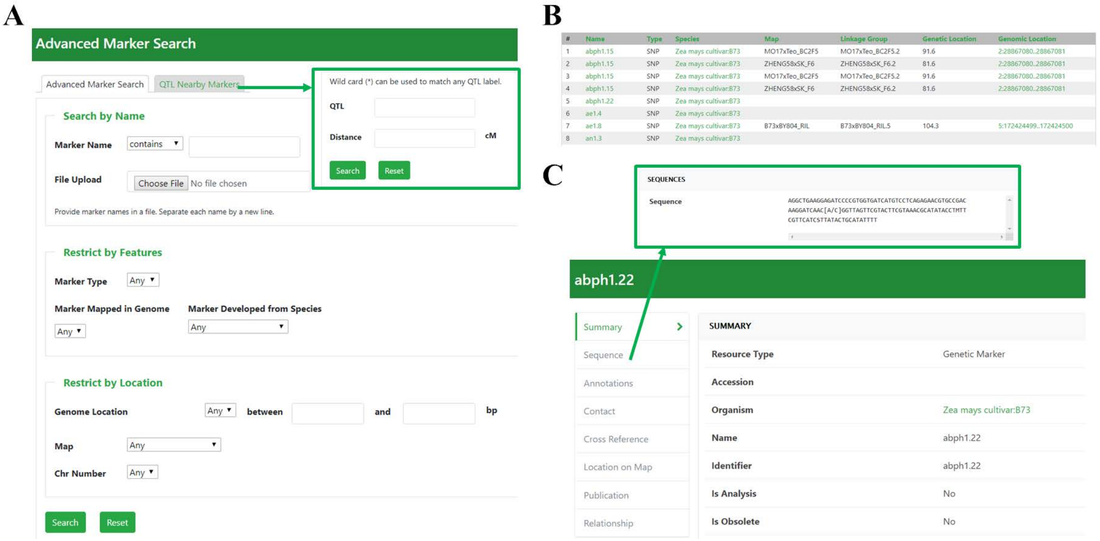

Figure S11. Genetic marker search functions in ZEAMAP, Related to Figure 3. The genetic markers in ZEAMAP could be searched by their names, features and locations through "Advanced Marker Search" function (A) or by their distances with certain QTL through "QTL Nearby Marker" function (inset in A). The search result lists general information of genetic and physical locations for each record, with physical locations linked to Jbrowse visualization (B). Click on each marker name would lead to the detailed page for this marker including the flanking sequence and the location on maps (C).

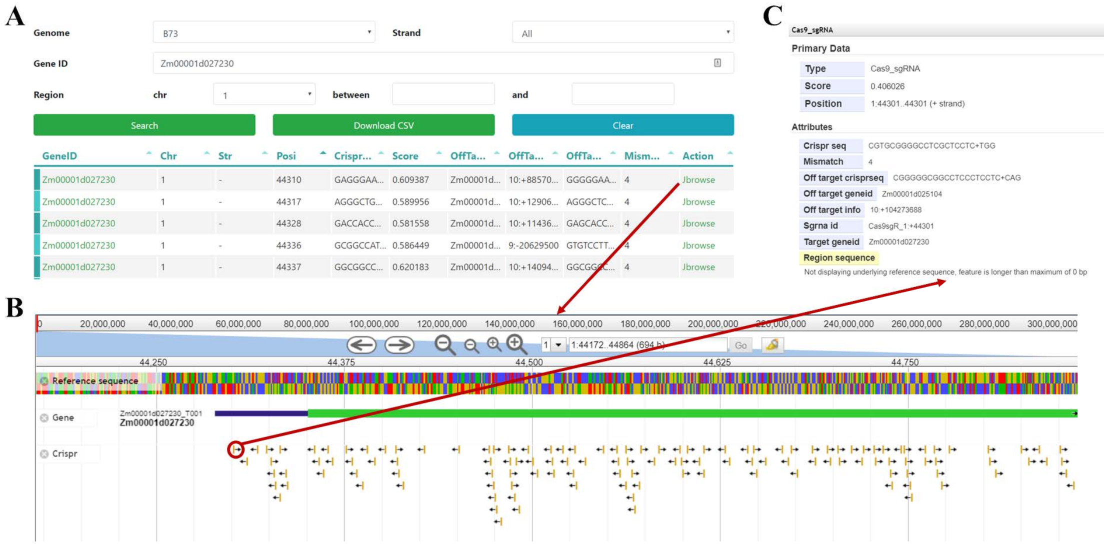

Figure S12. Crispr sgRNA function in ZEAMAP, Related to Figure 1. The sgRNA information could be searched by IDs and genome locations of target genes through table browser (A), the resulted records have links to their locations on Jbrowse (B) and the detail page of each Cas9_sgRNA element on Jbrowse shows the information of target and off-target genes (C).

Transparent Methods

Data collection

The pedigree information of elite inbred lines in the maize association mapping panel was collected from (Yang et al., 2011). The B73 reference genome assembly (AGPv4) and annotation files (Version 4.43) were downloaded from Gramene (https://www.maizegdb.org/genome/genome_assembly/Zm-B73-REFERENCE-GRAMENE-4.0), while the Mo17 reference genome (version 1.0) and annotations (version 1.0) were downloaded from MaizeGDB (https://ftp.maizegdb.org/MaizeGDB/FTP/Zm-Mo17-REFERENCE-CAU-1.0/). The SK reference genome (version 1.0), annotations (version 1.0) and RNA-seq data of nine SK tissues were collected from (Yang et al., 2019). The HZS genome assembly, annotations and RNA-sequencing data were retrieved from Genome Sequence Archive in Beijing Institute of Genomics (BIG) Data Center (http://bigd.big.ac.cn/gsa) with project ID PRJCA001247. The genome assembly and annotations of *Zea mays* ssp. *mexicana* were from (Yang et al., 2017). RNA-sequencing of different B73 tissues were from (Walley et al., 2016), and RNA-seq data of developing maize kernels from 368 AMP inbred lines were from (Li et al., 2013). The chromatin interaction and histone modification data were from (Peng et al., 2019), the chromatin accessibility data were from (Rodgers-Melnick et al., 2016), and the DNA methylation data of the AMP were from (Xu et al., 2019). Variants, GWAS and eQTL signals, and phenotypes of the AMP, including agronomic traits, kernel amino acid contents, kernel lipid contents and metabolomic data were collected from previously reported studies (Liu et al., 2015; Wen et al., 2014; Yang et al., 2014). Linkage maps and QTL mapping results were collected from (Pan et al., 2016).

Functional annotation

For each genome annotation, the protein sequences of the predicted genes were compared against the InterPro database using InterProScan 5 (Jones et al., 2014) to identify functional protein domains. The proteins were further compared against the GenBank non-redundant protein (nr) database using Basic Local Alignment Search Tool (BLAST) with the options “-p blastp –e 1e-05 –b 5 –v 5 –a 4 –m 7 –F F”. The BLAST results against the nr database and the Interpro results were further analyzed by Blast2GO (Conesa et al., 2005) to assign gene ontology (GO) terms. Kyoto Encyclopedia of Genes and Genomes (KEGG) annotations were performed by running BLAST against the KEGG database (version 84.0) with options “-p blastp -e 1e-05 -a 4 -m 8 -F F”. The proteins were also searched against PFAM version 32.0 (Finn et al., 2014) using HMMer 3.1b2 (Potter et al., 2018) with default parameters. To identify gene orthologs and clusters of orthologous group (COG) annotations, the proteins were mapped to eggNOG orthology database (version 4.5.1) (Huerta-Cepas et al., 2015) using emapper-1.0.3 (Huerta-Cepas et al., 2017). To add gene-product annotations, the proteins were searched against UniProt database (version 2019_04) (Consortium, 2018) using Diamond (v0.8.22.84) (Buchfink et

al., 2015) with the options "--evalue 1e-05 --max-target-seqs 1", the UniProt and EggNog search results were combined to get the gene and product names using Gene2Product v1.32 (https://github.com/nextgenusfs/gene2product). Possible proteolytic enzymes were annotated by searching the proteins against the MEROPS database (version 12.0) (Rawlings et al., 2017) using Diamond with the options "--evalue 1e-05 --max-target-seqs 1". The proteins were also searched against the embryophyta single copy ortholog models from BUSCO Datasets (embryophyta_odb9, update date: 2017-02-13) (Simao et al., 2015) using HMMer with default options.

Comparative genomics

To identify synteny blocks, we first compared proteins from one genome to those from another using BLASTP with an E-value cutoff of 1e-10 and a maximum number of alignments of 5. The significant hits were then analyzed by MCScanX (Wang et al., 2012) with parameters "-k 50 -g -1 -s 5 -e 1e-10 -m 25 -w 5" to obtain synteny blocks. The whole genome alignments between two genomes in ZEAMAP were performed using minimap2 (version 2.17-r941) (Li, 2018) , with parameters "-c -x asm5 -B5 -O4,16 --no-long-join -r 85 -N 50 -s 65 -z 200 --mask-level 0.9 --min-occ 200 -g 2500 --score-N 2", and the raw alignment results were filtered to get the best alignment for each contig with QUAST-LG (Mikheenko et al., 2018).

Annotating of genetic variations

The SNPs and InDels were annotated using the Ensembl variant effect predictor (VEP) (McLaren et al., 2016) according to B73 gene annotation v4.43. The polymorphic SVs between B73 and SK, as well as their genotypes in the AMP, were retrieved from (Yang et al., 2019), and annotated according to B73 gene annotation v4.43 using SURVIVOR v1.0.6 (Jeffares et al., 2017). Haplotype blocks and tag SNPs were identified using Haploview (Barrett et al., 2004).

Mapping and filtering of genetic loci

To perform genome-wide association studies for the collected phenotypic traits, the SNPs were then further filtered to keep only records with a minor allele frequency (MAF) of at least 5%. A mixed linear model accounting for the population structure (Q) and familial relationship (K) was used to examine the association between the SNPs and each trait using Tassel3 (Bradbury et al., 2007). The *P* value of each SNP was calculated, and significance was defined with Bonferroni corrected *P* value cutoff of 1/N, where N is the total number of markers used. To prevent the interactive GWAS viewers and the tabular loci browser from operating too slowly, the volume of GWAS results was reduced by filtering out SNPs which had very low significance values (*P* value > 1e-4). The pairwise LD r^2 values of the remaining SNPs for each trait within 500 Kb windows were calculated using PopLDdecay (Zhang et al., 2018).

We kept *cis*-eQTLs alone by retaining only the SNPs within 1 Mb of each gene (Lonsdale et al., 2013). High quality *cis*-eQTL SNPs were selected by only retaining those

with a *P* value smaller than the Bonferroni corrected *P* value cutoff of 1/N. The pairwise LD r^2 values of the remaining SNPs for each gene were calculated using PopLDdecay (Zhang et al., 2018).

Interactive visualization tools

The visualization tools for GWAS results were developed using LocusZoom.js (https://github.com/statgen/locuszoom), a JavaScript embeddable plugin for interactively visualizing statistical genetic data, and ECharts (https://www.echartsjs.com), an open-sourced JavaScript visualization tool. The gene expression pattern viewer and the eQTL visualizer were modified from GTEx visualizations (https://github.com/broadinstitute/gtex-viz) (Lonsdale et al., 2013). The principal component analyses (PCA) dot plot and the ancestries stacked histogram were also developed using ECharts.

CRISPR/Cas9 single-guide RNA designing

CRISPR/Cas9 sgRNAs for each maize reference genome were designed using CRISPR-Local (Sun et al., 2018) with default options. Results were converted into gff format with in-house perl scripts to format them for JBrowse.

Supplemental References

Barrett, J.C., Fry, B., Maller, J., and Daly, M.J. (2004). Haploview: analysis and visualization of LD and haplotype maps. Bioinformatics *21*, 263-265.

Bradbury, P.J., Zhang, Z., Kroon, D.E., Casstevens, T.M., Ramdoss, Y., and Buckler, E.S. (2007). TASSEL: software for association mapping of complex traits in diverse samples. Bioinformatics *23*, 2633-2635.

Buchfink, B., Xie, C., and Huson, D.H. (2015). Fast and sensitive protein alignment using DIAMOND. Nature Methods *12*, 59-60.

Conesa, A., Götz, S., García-Gómez, J.M., Terol, J., Talón, M., and Robles, M. (2005). Blast2GO: a universal tool for annotation, visualization and analysis in functional genomics research. Bioinformatics *21*, 3674-3676.

Consortium, T.U. (2018). UniProt: a worldwide hub of protein knowledge. Nucleic Acids Research *47*, D506-D515.

Finn, R.D., Bateman, A., Clements, J., Coggill, P., Eberhardt, R.Y., Eddy, S.R., Heger, A., Hetherington, K., Holm, L., Mistry, J.*, et al.* (2014). Pfam: the protein families database. Nucleic acids research *42*, D222-D230.

Huerta-Cepas, J., Forslund, K., Coelho, L.P., Szklarczyk, D., Jensen, L.J., von Mering, C., and Bork, P. (2017). Fast Genome-Wide Functional Annotation through Orthology Assignment by eggNOG-Mapper. Molecular Biology and Evolution *34*, 2115-2122.

Huerta-Cepas, J., Szklarczyk, D., Forslund, K., Cook, H., Heller, D., Walter, M.C., Rattei, T., Mende, D.R., Sunagawa, S., Kuhn, M.*, et al.* (2015). eggNOG 4.5: a hierarchical orthology framework with improved functional annotations for eukaryotic, prokaryotic and viral sequences. Nucleic Acids Research *44*, D286-D293.

Jeffares, D.C., Jolly, C., Hoti, M., Speed, D., Shaw, L., Rallis, C., Balloux, F., Dessimoz, C., Bähler, J., and Sedlazeck, F.J. (2017). Transient structural variations have strong effects on quantitative traits and reproductive isolation in fission yeast. Nature communications *8*, 14061.

Jones, P., Binns, D., Chang, H.-Y., Fraser, M., Li, W., McAnulla, C., McWilliam, H., Maslen, J., Mitchell, A., Nuka, G.*, et al.* (2014). InterProScan 5: genome-scale protein function classification. Bioinformatics *30*, 1236-1240.

Li, H. (2018). Minimap2: pairwise alignment for nucleotide sequences. Bioinformatics *34*, 3094-3100.

Li, H., Peng, Z., Yang, X., Wang, W., Fu, J., Wang, J., Han, Y., Chai, Y., Guo, T., Yang, N.*, et al.* (2013). Genome-wide association study dissects the genetic architecture of oil biosynthesis in maize kernels. Nature genetics *45*, 43.

Liu, H., Wang, X., Warburton, M.L., Wen, W., Jin, M., Deng, M., Liu, J., Tong, H., Pan, Q., Yang, X.*, et al.* (2015). Genomic, transcriptomic, and phenomic variation reveals the complex adaptation of modern maize breeding. Molecular plant *8*, 871-884.

Lonsdale, J., Thomas, J., Salvatore, M., Phillips, R., Lo, E., Shad, S., Hasz, R., Walters, G., Garcia, F., Young, N.*, et al.* (2013). The genotype-tissue expression (GTEx) project. Nature genetics *45*, 580.

McLaren, W., Gil, L., Hunt, S.E., Riat, H.S., Ritchie, G.R., Thormann, A., Flicek, P., and Cunningham, F. (2016). The ensembl variant effect predictor. Genome biology *17*, 122.

Mikheenko, A., Prjibelski, A.D., Saveliev, V., Antipov, D., and Gurevich, A. (2018). Versatile genome assembly evaluation with QUAST-LG. intelligent systems in molecular biology *34*, i142–i150.

Pan, Q., Li, L., Yang, X., Tong, H., Xu, S., Li, Z., Li, W., Muehlbauer, G.J., Li, J., and Yan, J. (2016). Genome–wide recombination dynamics are associated with phenotypic variation in maize. New Phytologist *210*, 1083-1094.

Peng, Y., Xiong, D., Zhao, L., Ouyang, W., Wang, S., Sun, J., Zhang, Q., Guan, P., Xie, L., Li, W.*, et al.* (2019). Chromatin interaction maps reveal genetic regulation for quantitative traits in maize. Nature communications *10*, 2632.

Potter, S.C., Luciani, A., Eddy, S.R., Park, Y., Lopez, R., and Finn, R.D. (2018). HMMER web server: 2018 update. Nucleic Acids Research *46*, W200-W204.

Rawlings, N.D., Barrett, A.J., Thomas, P.D., Huang, X., Bateman, A., and Finn, R.D. (2017). The MEROPS database of proteolytic enzymes, their substrates and inhibitors in 2017 and a comparison with peptidases in the PANTHER database. Nucleic Acids Research *46*, D624-D632.

Rodgers-Melnick, E., Vera, D.L., Bass, H.W., and Buckler, E.S. (2016). Open chromatin reveals the functional maize genome. Proceedings of the National Academy of Sciences *113*, E3177-E3184.

Simao, F.A., Waterhouse, R.M., Ioannidis, P., Kriventseva, E.V., and Zdobnov, E.M. (2015). BUSCO: assessing genome assembly and annotation completeness with single-copy orthologs. Bioinformatics *31*, 3210-3212.

Sun, J., Liu, H., Liu, J., Cheng, S., Peng, Y., Zhang, Q., Yan, J., Liu, H.-J., and Chen, L.-L. (2018). CRISPR-Local: a local single-guide RNA (sgRNA) design tool for non-reference plant genomes. Bioinformatics *35*, 2501–2503.

Walley, J.W., Sartor, R.C., Shen, Z., Schmitz, R.J., Wu, K.J., Urich, M.A., Nery, J.R., Smith,

L.G., Schnable, J.C., and Ecker, J.R. (2016). Integration of omic networks in a developmental atlas of maize. Science *353*, 814-818.
Wang, Y., Tang, H., DeBarry, J.D., Tan, X., Li, J., Wang, X., Lee, T.-h., Jin, H., Marler, B., Guo, H.*, et al.* (2012). MCScanX: a toolkit for detection and evolutionary analysis of gene synteny and collinearity. Nucleic acids research *40*, e49-e49.
Wen, W., Li, D., Li, X., Gao, Y., Li, W., Li, H., Liu, J., Liu, H., Chen, W., Luo, J.*, et al.* (2014). Metabolome-based genome-wide association study of maize kernel leads to novel biochemical insights. Nature communications *5*, 3438.
Xu, J., Chen, G., Hermanson, P.J., Xu, Q., Sun, C., Chen, W., Kan, Q., Li, M., Crisp, P.A., Yan, J.*, et al.* (2019). Population-level analysis reveals the widespread occurrence and phenotypic consequence of DNA methylation variation not tagged by genetic variation in maize. Genome biology *20*, 243.
Yang, N., Liu, J., Gao, Q., Gui, S., Chen, L., Yang, L., Huang, J., Deng, T., Luo, J., He, L.*, et al.* (2019). Genome assembly of a tropical maize inbred line provides insights into structural variation and crop improvement. Nature genetics *51*, 1052.
Yang, N., Lu, Y., Yang, X., Huang, J., Zhou, Y., Ali, F., Wen, W., Liu, J., Li, J., and Yan, J. (2014). Genome wide association studies using a new nonparametric model reveal the genetic architecture of 17 agronomic traits in an enlarged maize association panel. PLoS Genetics *10*, e1004573.
Yang, N., Xu, X.-W., Wang, R.-R., Peng, W.-L., Cai, L., Song, J.-M., Li, W., Luo, X., Niu, L., Wang, Y.*, et al.* (2017). Contributions of Zea mays subspecies mexicana haplotypes to modern maize. Nature communications *8*, 1874.
Yang, X., Gao, S., Xu, S., Zhang, Z., Prasanna, B.M., Li, L., Li, J., and Yan, J. (2011). Characterization of a global germplasm collection and its potential utilization for analysis of complex quantitative traits in maize. Molecular Breeding *28*, 511-526.
Zhang, C., Dong, S.-S., Xu, J.-Y., He, W.-M., and Yang, T.-L. (2018). PopLDdecay: a fast and effective tool for linkage disequilibrium decay analysis based on variant call format files. Bioinformatics *35*, 1786-1788.
